# The effect of temporal expectation on the correlations of frontal neural activity with alpha oscillation and sensory-motor latency

**DOI:** 10.1038/s41598-023-29310-8

**Published:** 2023-02-03

**Authors:** Joonyeol Lee

**Affiliations:** 1 Center for Neuroscience Imaging Research, Institute for Basic Science (IBS), Suwon, 16419 Republic of Korea; 2 Department of Biomedical Engineering, Sungkyunkwan University, Suwon, 16419 Republic of Korea; 3 Department of Intelligent Precision Healthcare Convergence, Sungkyunkwan University, Suwon, 16419 Republic of Korea

**Keywords:** Neuroscience, Cognitive neuroscience, Oculomotor system, Sensorimotor processing

## Abstract

In a dynamic environment, we seek to enhance behavioral responses by anticipating future events. Previous studies have shown that the probability distribution of the timing of future events could shape our expectation of event timing; furthermore, the modulation of alpha oscillation is known to be a critical neural factor. However, a link between the modulation of alpha oscillation by temporal expectation and single neural activity is missing. In this study, we investigated how temporal expectation modulated frontal neural activities and behavioral reaction time by recording neural activity from the frontal eye field smooth pursuit eye movement region of monkeys while they performed a smooth pursuit eye movement task. We found that the temporal expectation reduced the coherence between the neural spiking and alpha frequency of the local field potential, along with the trial-by-trial correlation between the neural spiking activity and pursuit latency. This result suggests that the desynchronization of alpha oscillation by temporal expectation would be related to the decorrelation of population neural activity, which could be the neural source of reaction time enhancement by temporal expectation.

## Introduction

When a sprinter is waiting for the starting signal, the sprinter develops an expectation of the timing of the signal and allocates attention to react to the sound as soon as possible. When the expectation matches the actual timing, the reaction time is faster than when the expectation does not match the timing. Similarly, in our daily lives, we often develop expectations to anticipate a certain future event, and previous studies have shown that human behavioral performance depends on the development of temporal expectation^1,2^. The developed temporal expectation itself is often relatively precise, as if the brain represents the actual passage of time and calculates the probability of event occurrence even though the event has not happened yet. Previous studies have shown that the modulation of behavioral performance and single neural activity indeed follow this hazard rate^3^. A seminal study by Ghose and Maunsell showed that the modulation of sensory neural responses in macaque area V4 followed the hazard rate of stimulus change timing^4^. Janssen et al. showed that macaque lateral intraparietal area (LIP) neural responses are modulated according to the probability of random wait time between target onset and ‘go’ signal in the delayed saccade task^5^. Additionally, alpha/gamma frequency oscillation and neural activity in the primary visual cortex are known to be modulated by temporal expectation^6,7^. These studies showed that the hazard rate and expectation of stimulus timing are represented in the neural responses of the visual and parietal cortical areas.

On the other hand, human EEG studies have suggested the importance of occipital alpha oscillation in temporal expectation. They consistently reported that temporal expectation improved behavioral responses by reducing the power of alpha oscillations^8–11^. Apparently, the anticipation of the future event through temporal expectation is one of our cognitive strategies for using our limited resources efficiently and modulating both behavioral performance and neural responses. Despite many studies reporting the role of alpha oscillation in temporal attention and expectation, information remains limited about how alpha modulation is linked to spiking activities. Additionally, most previous studies reported the functional role of temporal expectation in the parietal and occipital sensory cortical areas. Therefore, it is not clear whether the modulation of neural activity by temporal expectation is limited to sensory cortical regions.

In both human and animal studies, an important behavioral signature of temporal expectation is the modulation of reaction time. Therefore, neural mechanisms that control the behavioral reaction time would be tightly related to how temporal expectation modulates behavioral responses. In a smooth pursuit eye movement, neural mechanisms of reaction time have been studied through the investigation of the trial-by-trial correlation between neural activity and behavior. Recent studies showed that a substantial amount of behavioral reaction time variation in smooth pursuit eye movements could be explained by the correlated latency variation in the area MT^12^ and frontal eye field smooth eye movement (FEF_SEM_) region^13^. In FEF_SEM_, we found that a brief local field potential (LFP) wave of 5 ~ 15 Hz (which overlaps with a nominal alpha oscillation) demonstrated a strong relationship with the behavioral reaction time variation, which suggested that this component might represent a neural latency covariation in a local neural population^13^. Therefore, the desynchronization of alpha oscillation by temporal expectation may be related to the reduction in neural latency covariation. The reduction of neural latency covariation by temporal expectation subsequently might result in the reduced neuron-pursuit latency correlation because the correlated neural variation in each node of the smooth pursuit circuit would contribute to the behavioral variations^12–15^. In the smooth pursuit eye movement system, human and nonhuman primates can also develop a temporal expectation of motion onset timing; the temporal expectation was verified by showing that the timing of anticipatory eye movement follows the hazard rate^16^. In a typical smooth pursuit paradigm, the timing of target motion is randomized, following a particular probability distribution (typically, a uniform distribution) to prevent the subjects from anticipating the upcoming target motion. This experimental design naturally enables the subjects to develop a temporal expectation following the hazard rate shaped from the randomized onset timing of the pursuit target.

In this study, we took advantage of the temporal expectation shaped in the smooth pursuit eye movement paradigm and investigated its relationships with alpha oscillation and neural activity found in FEF_SEM_. Based on our previous result^13^, suggesting that the 5 ~ 15 Hz LFP wave (alpha oscillation) represents neural latency covariation in FEF_SEM_, we hypothesized that the temporal expectation would decorrelate the neural activity by desynchronizing the alpha oscillation. Then, the single neural activity should be desynchronized from the alpha oscillation by the temporal expectation, and the trial-by-trial correlation between single neural activity and pursuit latency should be reduced.

## Materials and methods

We trained two rhesus macaques on a step-ramp smooth pursuit task. The step-ramp smooth pursuit task was developed to minimize the occurrence of saccadic eye movements during the initiation of pursuit^17^. In this task, the pursuit target was displaced eccentrically (step) and started moving toward the center of the screen at a constant speed (ramp). Prior to the commencement of behavioral conditioning, we performed two separate surgeries under the sterile surgical protocol. In the first surgery, we implanted a stainless steel cylindrical head holder on the skull using titanium straps, screws, and dental acrylic. Simultaneously, we also implanted a stainless-steel recording chamber over the cross-section of the arcuate sulcus and central sulcus to obtain access to the anterior bank of the arcuate sulcus. After the animal fully recovered from the surgical procedure, we performed a second surgery for implanting a scleral search coil on the right eye to monitor eye movements. As soon as the animals recovered from the surgery, we controlled the water consumption gradually and began behavioral training. After they were well trained on the step-ramp pursuit task, we performed a craniotomy inside the chamber to obtain access to the FEF_SEM_ region. Experiments were performed at Duke University, and all methods were approved in advance by the *Institutional Animal Care and Use Committee* at Duke University. The methods conformed to the *National Institutes of Health Guide for the Care and Use of Laboratory Animals*. All animals were not sacrificed after the experiment and were maintained in healthy conditions. The data presented here were used in a previous study that analyzed other aspects of the data for different purposes^13^. The study is reported in accordance with ARRIVE guidelines.

### Experimental design and data acquisition

Visual stimuli were presented on a gamma-corrected 24-inch CRT color monitor (Sony Trinitron, GDM-FW900). The spatial resolution of the screen was 2304 by 1440 pixels, and the screen covered 44 by 29° of the visual field. The vertical refresh rate of the monitor was 80 Hz; therefore, the visual stimuli were updated every 12.5 ms. All the stimuli were presented on a gray background (29 cd/m^2^), covering a luminance range between 0 and 56 cd/m^2^. We monitored the eye position using the scleral search coil technique, where magnetically induced voltage changes related to the eye position and velocity signals were obtained from the implanted coil after the voltages were decomposed into horizontal and vertical components through a phase detection circuit. The eye velocity was obtained by differentiating and employing a high-pass filter on the eye position at a cutoff frequency of 25 Hz through an analog circuit. The horizontal and vertical eye position and velocity traces were sampled at 1 kHz and saved for offline analysis. In a daily recording, we introduced one to three single electrodes or tetrodes into FEF_SEM_ and recorded the action potential and LFP. We used quartz-insulated platinum-tungsten electrodes, and the impedance of a single electrode was 2~4 MΩ, whereas the impedance of the tetrode was 1~2 MΩ (Thomas Recording GmbH). The neural signals were first amplified by a Tetrode Mini-matrix system (Thomas Recording GmbH) with a gain of 19. The input impedance of the preamplifier was 1 GΩ, which was sufficiently large compared to the impedance of single electrodes or tetrodes. This guaranteed the distortion to be minimal for low-frequency LFP phases^18^. The recorded voltage signals were transmitted through high- and low-pass filters with cutoff frequencies of 150 Hz for spikes and 170 Hz for LFP, respectively. LFPs were digitized at a sampling rate of 2 kHz. Whenever recorded voltage signals crossed a predefined threshold, we digitized a 1.2 ms duration of the spike waveforms at a sampling rate of 40 kHz. In addition, these signals were saved for online and offline analysis. We isolated single neural activity online using a window discriminator or principal component analysis. We later performed a more accurate offline spike sorting procedure, which was composed of principal component analysis and visual inspection of spike waveforms. The sorted spikes were sampled at 1 ms temporal resolution, and we visually inspected the rasters of every neuron to spot apparent errors in spike sorting. All offline spike sorting was performed using a Plexon Offline Sorter (Plexon Inc.). Digitization, filtering, amplification, and online spike sorting were performed in a Plexon MAP system (Plexon Inc.).

When we found neurons that showed oculomotor responses, we tested whether the neuron was more responsive to a smooth pursuit or to a saccade. We ran a procedure where saccade and smooth pursuit trials were randomly interleaved. The saccade trials required the animals to make 15° amplitude saccadic eye movements in eight directions (45° intervals), where a 0.3° by 0.3° black circular dot was used as a saccade target. In pursuit trials, the animals were asked to track a random dot stimulus. Random dot patches were made from 120 spots inside a circular window with a 4° diameter. They had the same mean luminance with a gray background because we included equal numbers of black and white dots. The contrast of the dot patch defined by the luminance ratio between black and white dots was 100%. After a random duration (from 1100 to 1900 ms) of fixation on a fixation target (0.3° by 0.3° black circular dot), a random dot patch stimulus appeared at the center of the screen. Immediately after stimulus onset, the dots inside the circular window moved into one of eight directions (45° interval) with 15 deg/s speeds for 100 ms. Subsequently, the random dot patch moved in the same direction and speed with the pattern motion for 650 ms. We measured the saccade and pursuit direction tunings online to identify the preferred pursuit direction. We recorded the neural data if a given neural response was tuned to the direction of the smooth pursuit and proceeded into the second, main experiment. In the later offline analysis, we discarded any neuron that showed more pronounced responses to saccade than those to pursuit.

**Figure 1:**
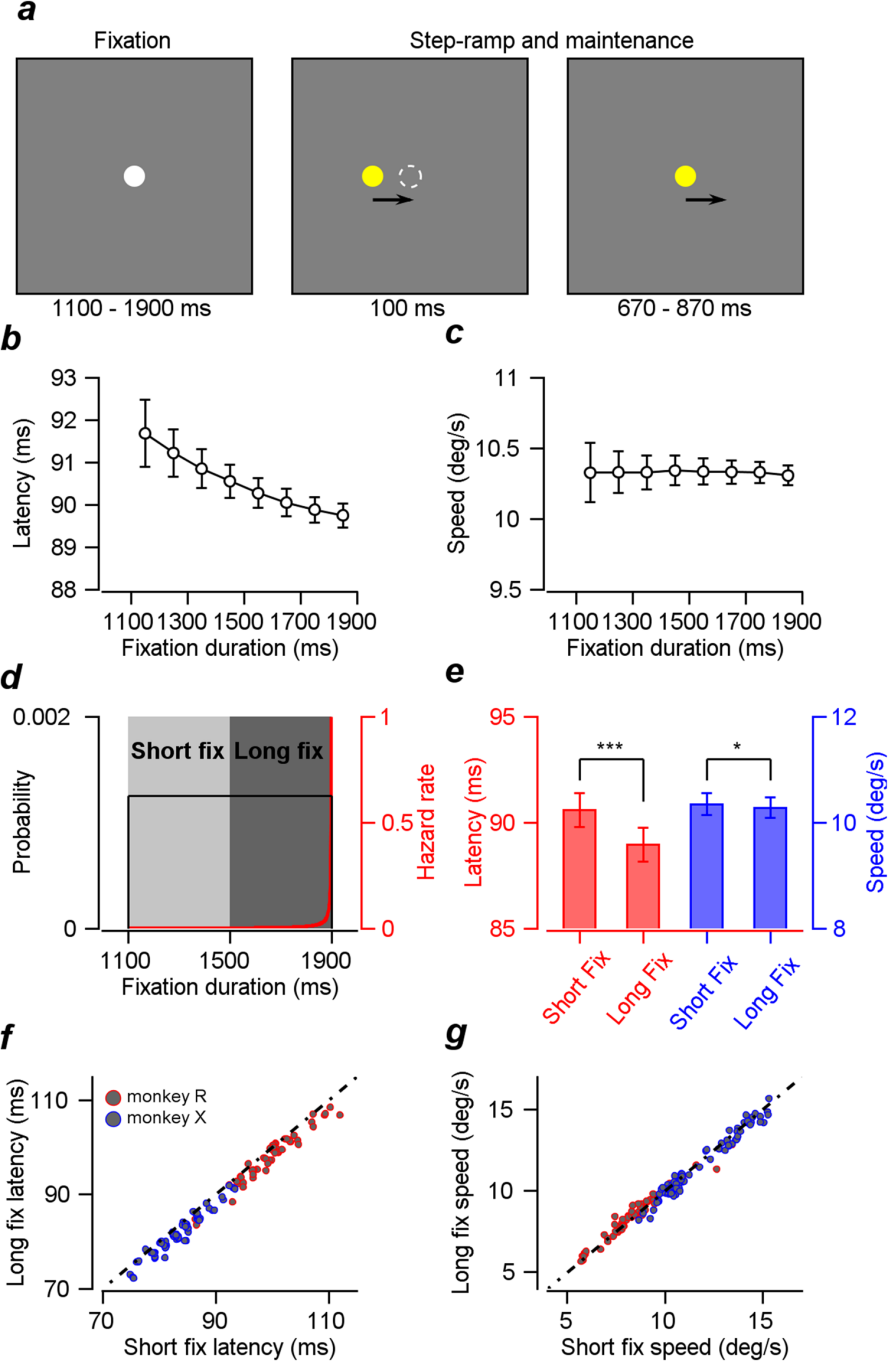
Experimental design and the effect of temporal expectation on smooth pursuit behaviors. (**a**) Temporal structure of the step-ramp smooth pursuit task. (**b**) The average pursuit latency as a function of the fixation duration. As the fixation duration increases, the latency decreases. Error bars are standard errors. (**c**) The average pursuit speed as a function of the fixation duration. The fixation duration did not modulate the pursuit speed. Error bars are standard errors. (**d**) The probability distribution of fixation duration and hazard rate. Light and dark gray shaded areas denote the two fixation duration conditions. (**e**) Average latency and speed differences in the two groups sorted by fixation duration. Both latency and speed differences were significant; however, the latency difference was more pronounced. Error bars are standard errors. (**f**) Scatter plot of mean pursuit latencies in short and long fixation duration conditions. The dotted line is the unity line. (**g**) Scatter plot of mean pursuit speeds in short fixation duration vs. those in long fixation duration conditions.

In the main experiment, the monkeys performed a step-ramp smooth pursuit eye movement task^17^. The overall experimental design appears in Fig. 1a. At the start of each trial, a white circular fixation point appeared at the center of the screen. After a random duration between 1100 and 1900 ms, a 0.6° by 0.6° yellow circular dot appeared as the pursuit target. The pursuit target appeared eccentrically to the center of the screen. Then, it started moving toward the center and continued to move at a constant speed for 770~870 ms. In each session, we chose two to four pursuit directions that provided reasonable compromises among the preferred directions of neurons under recording, along with one to two speeds (10°/s, 20°/s). The sizes of the steps in step-ramp pursuit were 1° and 3° for the two speeds.

The hazard rate in Fig. 1 was calculated following Eq. (1) below.

1 $$h\left( t \right) = \frac{p\left( t \right)}{{1 - F\left( t \right)}} = \frac{\frac{1}{800}}{{1 - \frac{1}{800}t}}$$

(*h*(*t*) is a hazard rate as a function of fixation duration, *p*(*t*) is a probability density function of an event occurring during the fixation duration, and *F*(*t*) is a cumulative probability density function).

### Estimation of pursuit latency

First, we discarded any trials that contained saccadic eye movements in the time window covering pursuit initiation (from − 100 to 210 ms from motion onset) through visual inspection. Notably, we applied a stricter criterion before the initiation of pursuit (0~100 ms from motion onset); therefore, any trial with microsaccades (more than 5 deg/s) was deleted. Subsequently, we used a method that was developed in a previous study to quantify the latency of pursuit initiation for each trial^19^. We computed the average horizontal and vertical eye velocities across all the desaccaded trials and used the averages between 20 ms before and 100 ms after pursuit initiation as templates. We rotated the whole pursuit traces such that the average pursuit initiation direction was equal to 45 degrees. Then, we shifted and scaled the templates for horizontal and vertical eye velocity in each trial and estimated the best match using the least-square method (we found the time shift and scaling parameters that minimized the sum of squared errors). For all the estimation procedures, we used the Opti Toolbox with the NOMAD algorithm^20^. We took the time shift as the pursuit latency in each trial. Next, we sorted all the trials by fixation duration and selected those trials whose fixation duration was shorter than 1500 ms as the low temporal expectation group. Other trials whose fixation duration was longer than 1500 ms were termed the high temporal expectation group. The effect of temporal expectation on the pursuit latencies was quantified by comparing the pursuit latencies of the two conditions.

### Trial-by-trial correlation between neural response and behaviors

For each neuron, we only selected one pursuit condition that provided the largest average response (between 51 and 150 ms from the mean neural response onset) across all directions and speed conditions tested on the given neuron. We simply took the trial-by-trial correlation between the spike count in a given time window (from − 100 to 300 ms relative to motion onset, with a step size of 5 ms and window size of ± 50 ms) and pursuit latency or speed. To obtain a robust correlation measure, we used Spearman’s rho.

### Local field potential analysis

We preprocessed the LFP data with a Butterworth filter to remove 60 Hz line noise using a function in the Fieldtrip MATLAB toolbox^21^. We used the Chronux MATLAB toolbox^22^ for all spectral analyses, including a spike-field coherence analysis and LFP power analysis. We used the Hanning window for the spectral analysis and zero padded it to interpolate the frequency values. The power and spike-field coherence were calculated from LFP and spike recordings in 300 ms duration on each given window. As per studies performed previously^13,19^, we first calculated the spike-field coherence and transformed it using the variance stabilization method^23^. To correct a potential confounder that can occur at low frequencies due to the transient responses^24^, we generated the “null distributions” of coherences across frequencies by shuffling trials. We compared the original variance stabilized coherences across frequencies with the null distributions calculated from 1000 shuffled samples and calculated the z scores as a function of frequency.

### Statistical test

To address the multiple comparisons problem in group comparisons for neural activities, neuron-pursuit latency correlations across time, LFP powers, and spike-field coherence across time and frequencies, we corrected the statistical criterion of the two-tailed, one-sample t test (it was always a one-sample test because a group comparison was performed within each cell or recording) with a nonparametric, cluster-based permutation approach using a statistical toolbox in MATLAB^25^. Under the assumption that temporally close or nearby frequency data points are correlated, significant time clusters or frequency clusters (or both) were selected when the p values of the successive t values were < 0.05. For each time or frequency cluster, the null distribution of the summed t values was calculated through random data shuffling with zeros (20,000 times) because we wanted to test if the time or frequency clusters of the group comparisons were significantly different from zero. The time or frequency clusters with corrected p values < 0.05 were considered statistically significant.

## Results

Two monkeys were trained on a smooth pursuit eye movement task (Fig. 1a, please see Materials and Methods for detail). We randomized the fixation duration in each trial following a uniform distribution, which made the monkeys develop a temporal expectation for the timing of the visual target motion because the hazard rate increased rapidly toward the maximum bound of the uniform distribution (1900 ms in this case, Fig. 1d).

We report three main findings that are distinct from our previous study^13^. First, temporal expectation modulates smooth pursuit latencies and FEF_SEM_ neural responses. Second, the trial-by-trial correlations between pursuit latencies and neural responses are significantly reduced as the temporal expectation strengthens. Third, the synchrony between the spiking activity and the local field potential is significantly reduced in the alpha frequency band when the temporal expectation is high.

### The effect of temporal expectation on smooth pursuit eye movements

We randomized the duration between the onset of the fixation stimulus and target motion following uniform distribution (Fig. 1a). This is primarily done to prevent subjects from anticipating the timing of the initiation of visual motion; however, the randomized duration also makes subjects develop temporal expectations for the motion onset. In this case, the conditional probability for target appearance and hazard rate increased steeply toward the maximum bound of the uniform distribution (Fig. 1d). Because the subjects’ expectations for motion onset follow a particular form of the hazard function^3,26^, their expectation for visual motion timing will be higher when they fixate for a longer duration than when they fixate for a shorter duration. To verify whether the fixation duration influences the initiation of smooth pursuit eye movement, we first sorted trials into seven groups by the fixation duration (from 1100 to 1900 ms in a 100 ms window size). The pursuit latency gradually decreased as the fixation duration increased, as if the temporal expectation had a negative relationship with reaction time; however, the pursuit speed did not change much (Figs. 1b,c). This result showed the expected relationship between temporal expectation and pursuit latency; it was not surprising because fixation duration was negatively correlated with pursuit latency in our previous study^13^. It is important to note that we used the hazard function as an indicator of subjective temporal expectation, not as a numerical predictor. As a result, the pattern of pursuit latency as a function of fixation duration did not match exactly with the shape of the hazard function (Fig. 1d).

To further quantify the effect of temporal expectation on pursuit initiation (and subsequently the relationship between neural activity and behavior), we divided the trials into two groups; one group with a fixation duration of less than 1500 ms (short fixation duration, Fig. 1d) and the other group with a fixation duration of more than 1500 ms (long fixation duration). Among a total of 141 experiments with different stimulus conditions (the number of trials was > 100 for each experiment), the effect of fixation duration on pursuit latency was significant in 69 experiments (two-sample t test, *p* < 0.05). Across all the sessions, the mean pursuit latencies in the short fixation duration condition were higher than latencies in the long fixation duration condition (Fig. 1e), which was statistically significant (90.58 ms vs. 88.94 ms, t test, within-subject design, *p* < 1.2 × 10^−30^, ${t}_{140}$ = 17.6). A comparison of the eye speeds between the two conditions showed a significant difference (t test, *p* = 0.033, ${t}_{140}$ = 2.154). Nevertheless, the average speed in the short fixation duration was slightly higher than that in the long fixation duration (10.34 deg/s vs. 10.28 deg/s). We reasoned that if the temporal expectation enhanced the pursuit speed, the speed in high temporal expectation would be higher; therefore, the pursuit speed would be higher in the long fixation duration condition than in the short fixation duration condition. Because the direction of the effect was opposite and the size was too small, we concluded that the influence of the temporal expectation on pursuit speed was minimal.

### The effect of temporal expectation on the responses of FEF_SEM_ neurons

**Figure 2:**
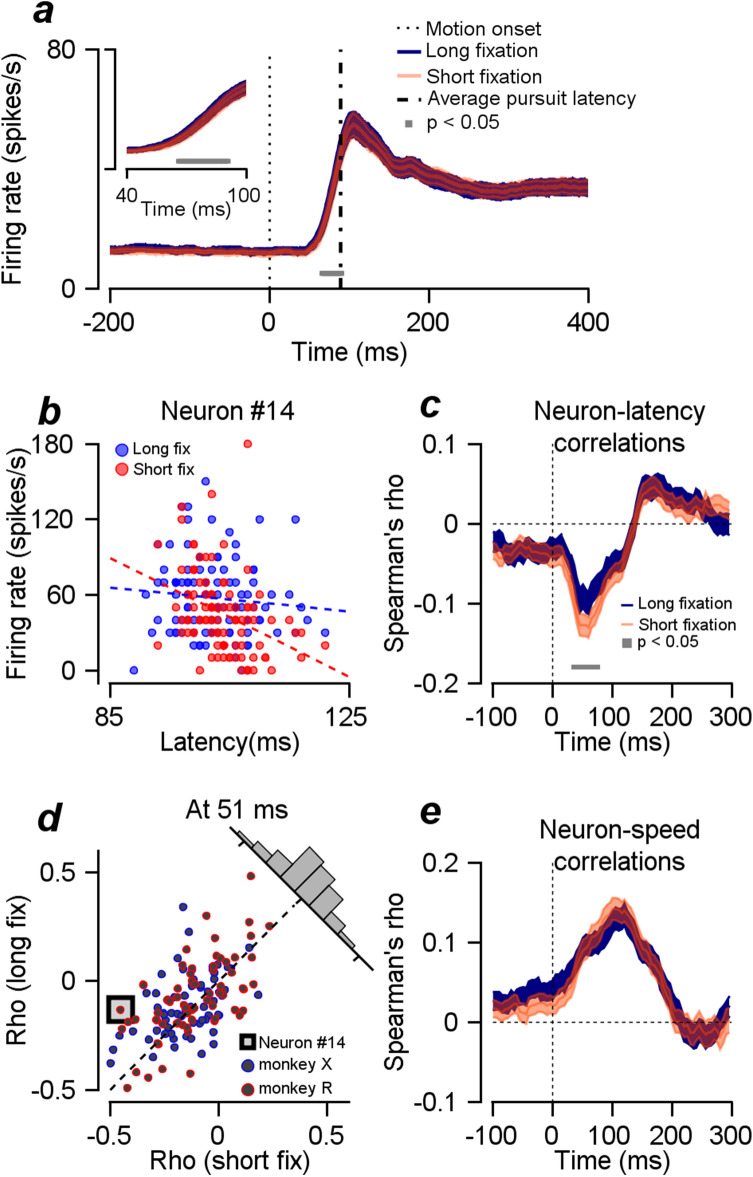
Effects of temporal expectation on FEF_SEM_ neural activities. (**a**) Effect of temporal expectation on the average PSTH of 133 neurons. The inset is a time-magnified figure. The gray bar shows the time points where PSTHs between the two conditions were significantly different from each other. The black dotted line shows the pursuit target onset timing, and the dash-dotted line shows the average pursuit latency. (**b**) A scatter plot of an example neural activity (neuron #14) showing the relationship between the pursuit latency and neural responses measured at 1~100 ms from motion onset. The trials were divided into two groups, one with a short fixation duration (fixation duration < 1500 ms) and the other with a long fixation duration (fixation duration > 1500 ms). (**c**) Average neuron-pursuit latency correlations for the two fixation duration conditions for 130 neurons as a function of time. The gray bar shows the time points of statistical significance. (**d**) A scatter plot of neuron-pursuit latency correlations comparing the two fixation duration conditions measured at 51 ms (a time window of 1~100 ms). The red contoured circles are from monkey R, and the blue contoured circles are from monkey X. The black contoured square shows the result of the example neuron. The inset histogram shows the distribution of correlation differences (short fixation duration–long fixation duration). (**e**) Average neuron-pursuit speed correlation for the two fixation duration conditions as a function of time.

Next, we observed the effect of fixation duration on the responses of the FEF_SEM_ neurons. Because the frontal eye field has been implicated for its role in attention, decision-making, and reaction time^27–32^, it is likely that the behavioral changes observed accompanied the changes in neural responses. Figure 2a shows the average peristimulus time histogram (smoothed with a rectangular time window with 20 ms size) of 133 FEF_SEM_ neurons (69 from monkey R, 64 from monkey X) for the two fixation duration conditions. A statistical test comparing the responses across the temporal expectation conditions showed sporadic significant differences in spontaneous and evoked activities (test result not shown). However, only the differences in the time duration between 66 and 91 ms from target onset remained significant when we corrected for multiple comparisons (cluster-based permutation test, cluster alpha = 0.05, test alpha = 0.05, Fig. 2a). This time duration may reflect the neural latency difference, and the higher firing rate in high temporal expectation may be because temporal expectation made neurons respond faster (we note that the direct estimation of neural latency using a previously established method^12,13^ did not reveal any significant differences across the fixation duration conditions, which could be due to the small effect size). Since we reported the correlation between FEF_SEM_ neural and behavioral latencies in the previous study^13^, the concurrent neural and behavioral latency changes by temporal expectation are not notable if temporal expectation is the source of the covariation; temporal expectation could have modulated behavioral latencies without directly affecting FEF_SEM_ neural activities. On the other hand, temporal expectation may enhance sensory-motor neural processes. Previous studies have demonstrated that cognitive factors, including attention and learning, can improve sensory processes by decorrelating population neural activities^33–36^. Additionally, the correlated neural activities in FEF_SEM_ contribute to pursuit variation, which provides the basis for the correlation between single neural activity and behavior^13,14^. Therefore, temporal expectation may enhance behavior by decorrelating neural activities (and latencies) in FEF_SEM_. Subsequently, the neuron-pursuit correlation will be modulated by the degree of temporal expectation, defined by fixation duration in our experiment.

To test this possibility, we analyzed the trial-by-trial correlation between FEF_SEM_ neural activities and pursuit initiation. In this analysis, we had to divide trials into two groups; therefore, we only included samples with more than 100 trials and firing rates higher than five spikes/s (at the time window between 50 and 150 ms from motion onset), which resulted in 130 out of 133 neurons. To prevent the requirements of the analysis method from dropping samples further, we calculated the spike count correlation instead of calculating neuronal latency-behavioral latency correlations^12,13^. Figure 2b shows the scatter plot of an example neuron, showing the correlation between the firing rate and pursuit latency in short and long fixation duration conditions. Due to the usage of a small time window (100 ms) for calculating neural responses, the firing rates (y-axis) were categorical, taking one of several values. This neuron showed a strong negative correlation in short fixation duration trials (Spearman’s rho = − 0.451, *p* = 2.4 × 10^−6^) and a weak negative correlation in long fixation duration trials (Spearman’s rho = − 0.132, *p* = 0.1767). When we observed the temporal progression of the population averages, the size of the negative correlation increased in a time window of approximately 31 and 100 ms from motion onset in both fixation duration trials (Fig. 2c). Given that the neural latency variation would be maximized in this time window, the negative correlation here may be due to the neuronal latency-behavioral latency correlations. In addition, the size of the negative correlation was significantly decreased when the temporal expectation was high (gray line, cluster-based permutation test, cluster alpha = 0.05, test alpha = 0.05, Fig. 2c, absolute values composing blue line were significantly smaller than those composing orange line). A snapshot of the correlation difference at the time point of 51 ms (1 ~ 100 ms) is presented in Fig. 2d (t test, within-subject design, *p* = 0.00688, ${t}_{129}$ = − 2.747). In our previous study, we reported the contribution of spontaneous activity to pursuit latency^13^. In this analysis, even if the spontaneous activity was negatively correlated with pursuit latency (the correlation in the time window − 100 m~0 ms, Fig. 2c), we could not find an effect of temporal expectation on the correlation between spontaneous activity and pursuit latency. The trial-by-trial correlations between FEF_SEM_ neural activities and pursuit speeds were positive, and peak correlation was observed at approximately 100 ms from motion onset. However, a correlation difference between the short and long fixation duration conditions was not observed (Fig. 2e).

### Temporal expectation reduces broadband oscillation in the local field potential

**Figure 3:**
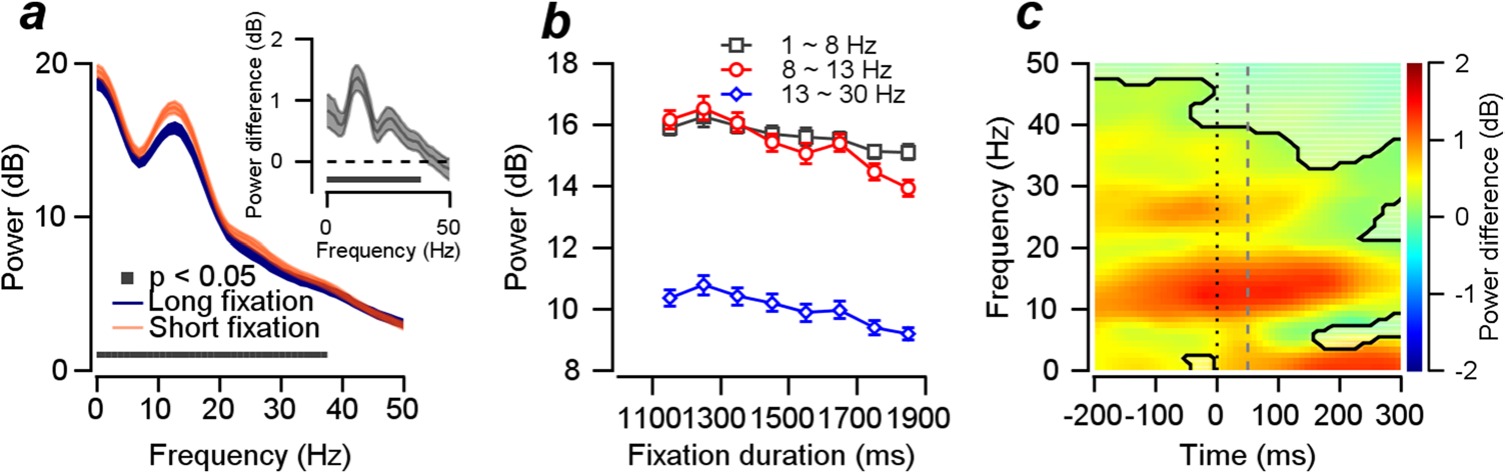
The effect of temporal expectation on the local field potential responses. (**a**) Average LFPs across 120 recordings in two fixation duration trials, estimated at a time window centered at 51 ms. Orange denotes the averaged power for low temporal expectation trials, and blue denotes the high temporal expectation trials. Colored areas show the standard errors. The inset shows the power difference. (**b**) Average LFP powers in three frequency groups plotted as a function of fixation durations. The error bars denote standard errors. (**c**) The LFP power difference between the fixation duration trial groups measured at time windows covering the duration of − 200 ms to 300 ms from motion onset. The black contour denotes statistically significant time and frequency regions (cluster-based permutation test, alpha = 0.05). The black dotted line shows motion onset timing, and the gray dashed line shows the time window for the data in Fig. 3a.

Notably, the neuron-pursuit latency correlations are modulated by temporal expectation. In our previous study, we showed that the 5~15 Hz frequency component of LFP has a tight relationship with the correlated neural latencies of the FEF_SEM_ local population^13^. Among potential possibilities that can explain the reduction of neuron-pursuit latency correlation, the most likely explanation would be the desynchronization of the neural activity from the correlated population^12–14,19,37–39^. Therefore, we hypothesized that the reduced neuron-pursuit latency correlation might be explained by the changes in synchrony between spikes and the alpha frequency of LFP. To verify this hypothesis, we first tested whether the strength of the neural oscillation in the alpha frequency changes according to the fixation duration conditions. If the reduced neuron-behavior correlation is due to the decorrelation of neural population latencies, the strength of the alpha frequency oscillation might be reduced when the temporal expectation is high. Figure 3a (also please see Supplementary Fig. 1a,b for frequency ranges up to 100 Hz) shows the spectral analysis of LFPs across 120 recordings estimated at a time window centered at 51 ms (− 100~200 ms) from the motion onset. Overall, there was a clear peak in the spectral power in alpha frequency oscillation (centered at 10 Hz) in both fixation duration conditions. The power in broadband (1~40 Hz) oscillation was significantly lower in high temporal expectation trials than in low temporal expectation trials (cluster-based permutation test, cluster alpha = 0.05, test alpha = 0.05). To verify whether the power of the low-frequency oscillation was reduced as a function of fixation duration, similar to the decrease in smooth pursuit latency (Fig. 1b), we divided the low-frequency oscillation into three ranges (1~8 Hz, 8~13 Hz, and 13~30 Hz) and plotted them as a function of fixation duration. We found that the powers of each frequency range oscillation decreased as the fixation duration increased (Fig. 3b), and the decrease was the steepest in alpha frequency (8~13 Hz) oscillation. To test if the power difference between the two fixation duration groups was maintained across time, we compared the oscillatory powers in two groups of fixation duration trials, from the time window centered at − 200 ms (− 350~− 50 ms) to the time window centered at 300 ms (150~450 ms) in 10 ms intervals. Although the largest difference was observed in alpha frequency oscillation, significant differences were observed across different times in broadband oscillations (Fig. 3c, cluster-based permutation test, cluster alpha = 0.05, test alpha = 0.05, significant regions are shown with black contours; please see Supplementary Fig. 2a,b for LFP powers for individual fixation duration groups).

### Temporal expectation reduces spike-field coherence in alpha frequency oscillation

**Figure 4:**
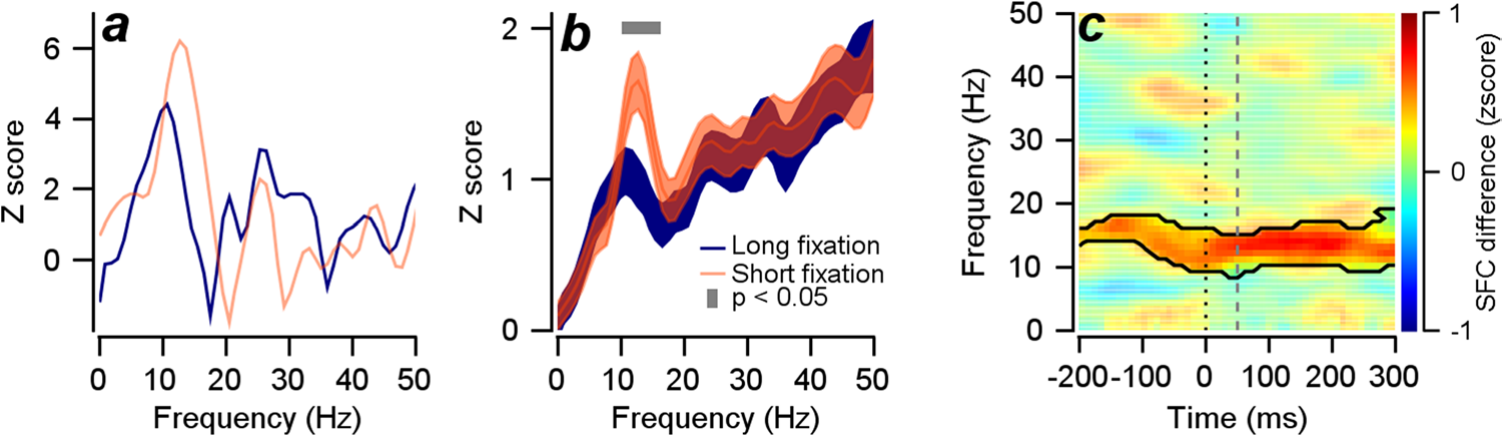
A spike-field coherence analysis. (**a**) Z scored spike-field coherence analysis for an example neuron. Orange color denotes the short fixation duration condition, and blue denotes the long fixation duration condition. (**b**) Z scored spike field coherence analysis for the 130 neurons. The gray rectangle shows the frequency when the two conditions are significantly different at the *p* value of 0.05. The shaded areas show the standard errors. (**c**) Z scored spike-field coherence analysis for the 130 neurons across different times. The black contour shows the significant time and frequency where zSFC in the short fixation duration was significantly higher than zSFC in the long fixation duration (cluster-based permutation test, *p* = 0.05). The black dotted line shows the timing for motion onset, and the gray dashed line shows the time window for the data in Fig. 4b.

The effect of temporal expectation (fixation duration) on the oscillatory power was not specific to the narrow band neural oscillation. We initially hypothesized that the alpha frequency oscillation would represent the population-level neural synchrony for behavioral latency. If this hypothesis is true, the temporal expectation will desynchronize the spiking activity only from the alpha frequency of LFP. To examine this possibility, we analyzed the synchrony between spikes and LFP using z scored spike-field coherence (zSFC) following the method used in our previous study (see Materials and Methods,^19^). Figure 4a shows the z scored spike-field coherence for an example neuron, measured in a time window centered at 51 ms (from − 100 to 200 ms from motion onset). When the temporal expectation is low (shorter fixation duration), zSFCs in the low frequency range, including the alpha frequency component, were impressively higher; however, they were not modulated much at higher frequencies. Across 130 neurons, we observed a significant reduction in zSFC in high temporal expectation trials only in alpha frequency ranges (Fig. 4b, 10~15 Hz, cluster-based permutation test, cluster alpha = 0.05, test alpha = 0.05) among neighboring frequencies that showed significant power reduction. Moreover, to test whether this desynchronization of spiking from alpha oscillation was consistently maintained across different time points, we calculated the zSFC difference between the two fixation duration groups, from the time window centered at − 200 ms to the time window centered at 300 ms in 10 ms intervals. Repeatedly, the desynchronization consistently appeared around alpha oscillation (Fig. 4c, cluster-based permutation test, cluster alpha = 0.05, test alpha = 0.05, black contour, please see Supplementary Fig. 2c,d for zSFCs in individual fixation duration conditions, and Supplementary Fig. 1c,d for frequency ranges up to 100 Hz).

## Discussion

In this study, we showed that temporal expectation modulated the relationship between FEF_SEM_ neural activity and smooth pursuit latency. Temporal expectation also modulated the relationship between neural activity and alpha-frequency LFP oscillations. This result suggests that temporal expectation might enhance pursuit behavior by decorrelating and desynchronizing population-level FEF_SEM_ activities (and latencies), which was shown by the reductions in neuron-pursuit latency correlation and spike-field coherence in alpha frequency oscillations.

### Modulation of neural activities and local field potential

Only a few studies have observed the effect of temporal expectation on neural responses in areas V4 and LIP^4,5^. They changed the hazard rate of the visual target (target onset, change onset, etc.) and showed that the neural responses were modulated following the hazard rate, implicating that the conditional probability of the timing of target onset is represented in the neural responses in areas V4 and LIP. Leveraging these results, we hypothesized that the monkeys’ subjective temporal expectation for visual motion timing would follow the hazard rate. Additionally, it will be high when the fixation duration is long because the visual motion onset timing follows a uniform distribution in our experiments. We confirmed the effect in the latency of smooth pursuit eye movements. Consistent with previous studies, the reaction time has an approximately inverse relationship with the hazard function; the pursuit latency was short when the hazard rate was high, and the latency was long when the hazard rate was low. Our results showed that temporal expectation only influences the latencies of pursuit initiation without notably changing other aspects of the behavior (Fig. 1e).

FEF_SEM_ neural activity was also modulated by temporal expectation. A significant effect existed, although it was small. Because the primary purpose of this study was to identify the relationships among temporal expectation, FEF_SEM_ spiking activity, and LFPs, which are more important in understanding the neural mechanisms than the neural response modulation itself, we did not focus on the spiking activity alone. In our samples, the effect was shown as neural response changes at response initiation (which might be explained mainly by neural latency changes). Previous studies have shown sustained neural response modulation by the hazard rate rather than just changes in neural response initiation. The difference between this study and previous studies might originate from differences in the experimental design. We did not present any visual stimulus in the visual receptive field^4^ or response field^5^ of a given neuron during the fixation duration except for the fixation point. This made FEF_SEM_ neurons remain relatively silent. Other studies reported an elevated spontaneous activity of FEF neurons toward saccade onset or smooth pursuit onset, which was more pronounced than what we observed here. Those studies typically let the subjects decide when to saccade^32^ or anticipate the speed of the upcoming tracking target^40,41^. In those cases, spontaneous activity naturally represented the decision processes or prediction of a feature rather than temporal expectations.

We found a striking modulation of LFP power at a frequency of approximately 10 Hz, which overlapped with the alpha frequency range. Previous EEG studies have shown that alpha-band EEG activity is related to the allocation of attention, working memory, and temporal expectation^8,10,11,42–47^. When the attentional load is high, the strength of the alpha band oscillation is attenuated. The reduction of the alpha oscillation by attention and temporal expectation reported previously matched well with what we observed here except for the reported brain regions. Most of the EEG studies found lateralized alpha modulation in the occipital regions of the brain. On the other hand, some previous studies reported the task-dependent reduction of alpha oscillation in FEF^48^ and temporal expectation-induced reduction of the central alpha and beta oscillation in a motor-oriented task^49^, which were consistent with our findings. Furthermore, studies have shown the top-down modulation of posterior alpha oscillation originating from frontal activity^50,51^. Therefore, frontal cortical modulation of alpha oscillations might be a controlling source of occipital alpha modulation. Alternatively, the modulation of the sensory cortical alpha oscillation^7^ may work as an input to the frontal alpha oscillation that we reported here, which would be consistent with the current understanding of the smooth pursuit eye movement circuit^52^.

### Relationships among FEF_SEM_ neural activity, alpha frequency oscillation, and behavioral latency

In our previous study, we showed that a 5~15 Hz frequency LFP wave in FEF_SEM_ is closely related to the correlated neural latencies. Only neurons with a robust synchronization with the LFP wave have a solid neural latency-pursuit latency correlation and neuron-neuron latency correlation^13^. If this is true, any changes in the synchronization between a 5~15 Hz wave and spike across trials should be linked to the changes in the neuron-pursuit latency correlation. Our results supported this view in the current study and provided insight into how the modulations of behavioral reaction time and the alpha oscillation by the temporal expectation were interrelated with each other. In a given session, when neural activity is synchronized with the alpha frequency of LFP oscillation because of low temporal expectation, the neuron-pursuit latency correlation becomes high. However, when neural activity is less synchronized with the alpha frequency of the LFP, the neuron-pursuit latency correlation becomes low. This relationship cannot be explained by simple changes in the power of LFP. In our observations, the LFP power changed by the temporal expectation occurring in broader frequency ranges, from 1 to 40 Hz. If the modulation of synchrony between the spikes and LFP is the passive result of power reduction, synchrony change should occur at all the above frequencies. The alpha frequency component might be unique and reflect the neural covariation^13^ that is linked to the smooth pursuit latency because the modulation of SFC only happens in the alpha frequency range. Therefore, as depicted in our previous work, we conclude that when single neural responses are synchronized with the alpha frequency of the LFP component, it is an essential factor in the size of the neuron-pursuit latency correlation (since the alpha frequency LFP is likely to represent the population level correlation between neurons). The temporal expectation may desynchronize the correlated population; as a result, the power of this component could have been reduced. Single neural activity was also desynchronized from this component, which was evident from the reduction of spike-field coherence in the alpha frequency by the temporal expectation. This result suggests that frontal cortical alpha oscillation may represent the correlation in neural responses, probably latencies, and temporal expectation is likely to enhance our behavioral reaction by desynchronizing the alpha oscillation along with reducing the neuron-pursuit latency correlation. In this interpretation, however, it is still uncertain why there is a persistent synchrony between spikes and the alpha frequency LFP after pursuit initiation. A possible explanation is that the alpha frequency SFC could reflect persistent neural and behavioral states brought on by temporal expectation. The cognitive states resulting from temporal expectation could persist throughout the trial and contribute to the persistent differences in SFC.

The findings from our study, in conjunction with previous research examining the relationship between neural covariations and behavioral responses^12–14,19,33,34,36,37,39^, suggest that the decreases in alpha synchrony and reaction time due to temporal expectation may be attributed to a decrease in neural covariation that is specific to behavioral reaction time. Despite this, several questions remain unresolved, and making a definitive conclusion based on correlational relationships is not feasible. Hence, further research is necessary to gain a more comprehensive understanding of this connection.

## Supplementary Information

Supplementary Figures.

## Data Availability

The datasets and codes used in the current study are available from the corresponding author on reasonable request.
