# Supplementary material for: The effect of temporal expectation on the correlations of frontal neural activity with alpha oscillation and sensory-motor latency
Source: Sci Rep. 2023 Feb 3;13:2012. doi: 10.1038/s41598-023-29310-8 (PMC9898494; doi:10.1038/s41598-023-29310-8)
Supplement: Supplementary file 1 — Supplementary Figures. [file 41598_2023_29310_MOESM1_ESM.docx]

**The effect of temporal expectation on the correlations of frontal neural activity with alpha oscillation and sensory-motor latency**

**Joonyeol Lee^1,2,3^**

^1^Center for Neuroscience Imaging Research, Institute for Basic Science (IBS), Suwon 16419, Republic of Korea

^2^Department of Biomedical Engineering, Sungkyunkwan University, Suwon 16419, Republic of Korea

^3^Department of Intelligent Precision Healthcare Convergence, Sungkyunkwan University, Suwon 16419, Republic of Korea


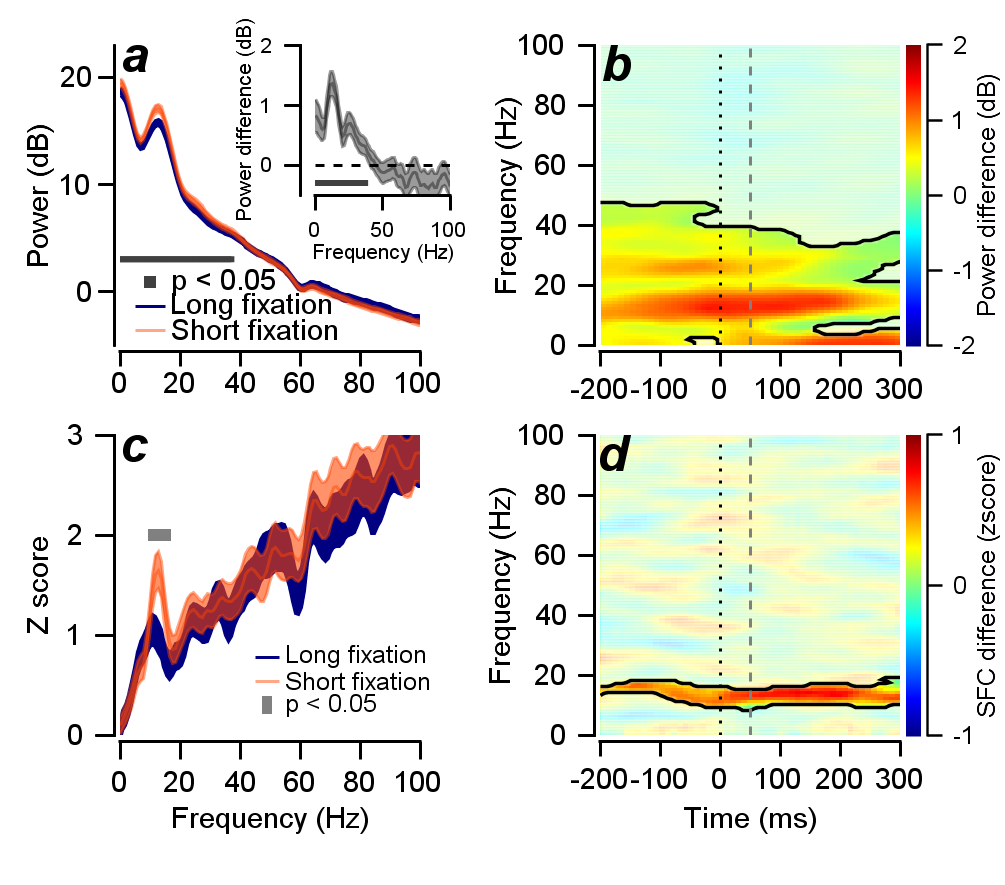


Supplementary Figure 1. The effect of the temporal expectation on the local field potential responses and the spike-field coherences. a: Average LFPs across 120 recordings in two fixation duration trials, estimated at a time window centered at 51 ms. Orange denotes the averaged power for low temporal expectation trials, and blue denotes the high temporal expectation trials. Colored areas show the standard errors. The inset shows the power difference. b: The LFP power difference between the fixation duration trial groups measured at time windows covering the duration of -200 ms to 300 ms from motion onset. The black contour denotes statistically significant time and frequency regions (cluster-based permutation test, alpha = 0.05). The black dotted line shows the motion onset timing, and the gray dashed line shows the time for Figure 3a. c: Z scored spike field coherence analysis for the 130 neurons. The gray rectangle shows frequency when the two conditions are significantly different at the p-value of 0.05. The shaded areas show the standard errors. d: Z scored spike-field coherence analysis for the 130 neurons across different times. The black contour showed the significant time and frequency where zSFC in the short fixation duration was significantly higher than zSFC in the long fixation duration (cluster-based permutation test, p = 0.05). The black dotted line shows the motion onset timing, and the gray dashed line shows the time for Figure 4b.


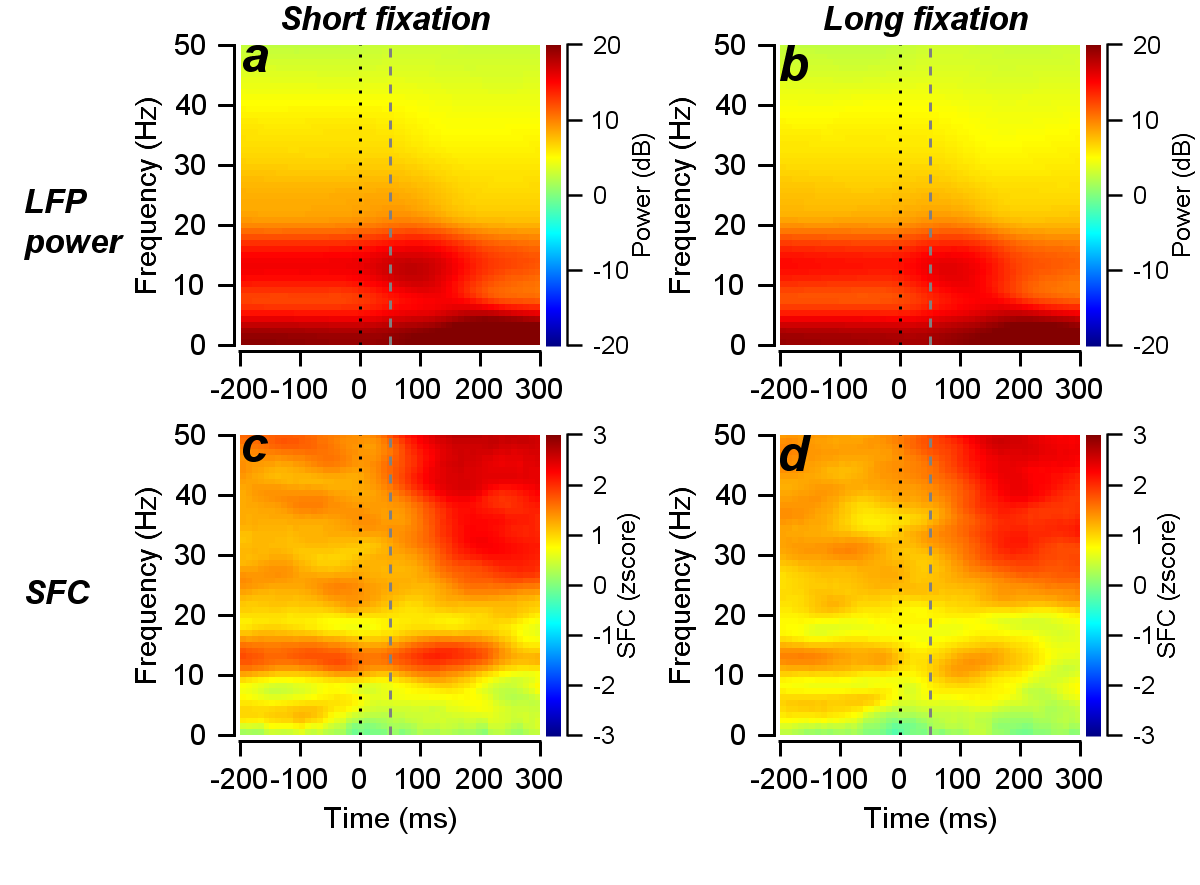


**Supplementary Figure 2. LFP power spectra and z-scored spike field coherences in individual fixation duration conditions. a:** LFP power spectra in short fixation duration condition. **b:** LFP power spectra in long fixation duration condition. **c:** Z scored spike field coherences in short fixation duration condition. **d**: Z scored spike field coherences in long fixation duration condition. The black dotted lines show the motion onset timing, and the gray dashed lines show the time for Figure 3a (Supplementary Figures 2a and b) and Figure 4b (Supplementary Figures 2c and d).
